# Probiotic Combination of *Lactiplantibacillus plantarum* M1 and *Limosilactobacillus reuteri* K4 Alleviates Early Weaning-Induced Intestinal Injury in Lambs via Modulation of Oxidative and Inflammatory Pathways

**DOI:** 10.3390/antiox15010132

**Published:** 2026-01-20

**Authors:** Qicheng Lu, Peng Zhang, Yujie Niu, Chuying Wang, Fengshuo Zhang, Junli Niu, Weibin Zeng, Cheng Chen, Wenju Zhang

**Affiliations:** 1 College of Animal Science and Technology, Shihezi University, Shihezi 832000, China; 20212313306@stu.shzu.edu.cn (Q.L.); 20222313401@stu.shzu.edu.cn (P.Z.); 20202313014@stu.shzu.edu.cn (Y.N.); 20232313201@stu.shzu.edu.cn (C.W.); zhangfengshuo@stu.shzu.edu.cn (F.Z.); niujunli@shzu.edu.cn (J.N.); zwbdky@shzu.edu.com.cn (W.Z.); 2 Bingtuan Key Laboratory for Efficient Utilization of Non-Grain Feed Resources, Shihezi University, Shihezi 832000, China

**Keywords:** early weaning, oxidative stress, inflammation, intestinal barrier function, Th17 cell differentiation, PPAR signaling

## Abstract

Early weaning in intensive lamb production improves reproductive efficiency but predisposes lambs to diarrhea, oxidative stress, and intestinal barrier dysfunction, highlighting the need for non-antibiotic strategies to protect gut health. This study evaluated whether a sheep-derived mixed probiotic could alleviate early weaning–induced intestinal injury and clarified its potential molecular mechanisms. Early weaning reduced body weight, average daily gain and feed efficiency, increased diarrhea, decreased plasma and colonic catalase (CAT), glutathione peroxidase (GSH-PX), and superoxide dismutase (SOD) activities, increased malondialdehyde (MDA), elevated interleukin-1β (IL-1β) and tumor necrosis factor-α (TNF-α), reduced interleukin-10 (IL-10) and transforming growth factor-β (TGF-β), increased plasma and mucosal immunoglobulin A, M, and G (IgA, IgM, IgG), and increased colonic lipopolysaccharide (LPS) with reduced diamine oxidase (DAO). Intestinally, EW induced villus atrophy, deeper crypts, lower villus height-to-crypt depth ratios, goblet cell loss, higher histopathological scores, and decreased colonic mucin 2, zonula occludens-1, claudin-1, and occludin. Probiotic supplementation partially reversed these alterations, restoring antioxidant enzyme activities, improving villus architecture and barrier protein expression, and rebalancing cytokine and immunoglobulin profiles. Transcriptomic and network analyses showed that early weaning activated Cytokine–cytokine receptor, NF-κB, TNF and Th17 pathways, whereas probiotics suppressed a weaning-responsive inflammatory gene module, downregulated key hub genes, and enhanced peroxisome proliferator-activated receptor (PPAR) signaling. These results show that supplementing early-weaned lambs with a mixed probiotic generated from sheep is an efficient nutritional strategy to reduce intestinal oxidative and inflammatory damage associated with weaning and to enhance their health and performance.

## 1. Introduction

Early weaning of lambs in intensive production systems can increase ewe reproductive efficiency and productivity while reducing feeding costs [1]. However, in practical flock management, diarrhea remains a major and persistent challenge that severely compromises production efficiency and economic returns [2]. This is particularly evident during the first two weeks after birth and after weaning, when the risk of diarrhea-associated mortality in lambs is markedly increased [3]. In early life, the immaturity of the immune system and the incomplete establishment of a stable intestinal microbiota render the lamb gut more susceptible to pathogenic invasion, thereby predisposing to diarrhea. Weaning is also accompanied by pronounced physiological and environmental stress; during this period, lambs experience multiple challenges, including changes in social grouping and alterations in diet composition and physical form [4], which increase intestinal exposure to pathogens and raise the risk of reactive oxygen species (ROS)-mediated damage to intestinal tissues [5,6]. Excessive ROS can induce DNA damage, protein aggregation, and dysfunction of cell membranes, ultimately leading to injury and apoptosis of gastrointestinal epithelial cells [7]. When the accumulation of ROS in the intestine exceeds the antioxidant defense capacity of the host, the resulting imbalance triggers oxidative stress [8]. Intestinal oxidative stress further promotes inflammation, impairs barrier function, and alters intestinal architecture [9,10], ultimately leading to a higher incidence of diarrhea and impaired growth and development. Therefore, alleviating intestinal oxidative stress during the weaning period and preserving the structural and functional integrity of the gut are critical for mitigating weaning stress and reducing the risk of diarrhea in lambs.

The intestine is not only the primary site for digestion and nutrient absorption but also the largest immune organ of the body [11]. Tight junctions between intestinal epithelial cells form the first mechanical barrier, limiting the entry of pathogens and toxins into the circulation. Studies have shown that early weaning markedly reduces the expression of tight junction proteins such as zonula occludens-1 (ZO-1) and claudin-1, thereby compromising intestinal barrier function [12]. Furthermore, Li et al. (2018) reported that alterations in the expression of genes related to intestinal barrier function in lambs can persist for up to 84 days during the weaning period [13], indicating that weaning stress exerts long-lasting effects on the intestinal barrier. Mucins secreted by goblet cells together with antimicrobial proteins produced by epithelial cells form the intestinal mucus layer [14]. This mucus layer prevents pathogens from colonizing the intestinal surface [15]. Weaning stress induces crypt hyperplasia and goblet cell loss in the colon, thereby disrupting the integrity of the mucus barrier [16]. Intestinal epithelial cells can also directly recognize both pathogenic and beneficial microorganisms and orchestrate immune responses, primarily through pattern recognition receptors (PRRs) [17]. Evidence from early-weaned goats demonstrates that signaling pathways involved in chemokine signaling, IL-17 signaling, and peroxisome proliferator-activated receptor (PPAR) activation are upregulated in the colonic mucosa, reflecting alterations in mucosal immune status [12]. In addition, the intestinal epithelium transports immunoglobulin A (IgA) into the lumen via the polymeric immunoglobulin receptor (pIgR) and responds to biological stress by secreting antimicrobial peptides, cytokines, and chemokines [18]. After weaning, mRNA expression of IL-6 and TNF-α increases in the jejunum and colon, whereas IL-10 expression decreases [19], suggesting an enhanced pro-inflammatory response and attenuated anti-inflammatory regulation. Cui et al. (2018) observed that, even on day 15 after weaning, the expression of proteins involved in immune processes remained significantly downregulated in the jejunal epithelium of lambs [20], further indicating that weaning stress has medium- to long-term effects on intestinal immune function.

As an important alternative to antibiotic therapy for intestinal inflammation, microecological preparations act by activating the intestinal immune system, promoting intestinal development, and alleviating oxidative stress, thereby reducing the incidence of diarrhea and supporting host growth and development. Probiotics have been shown to exert beneficial effects on intestinal health during the weaning period. Studies indicate that probiotics can improve intestinal barrier function and enhance antioxidant capacity through multiple mechanisms, thereby effectively reducing the occurrence of diarrhea [21,22]. On the one hand, probiotics modulate gut-associated lymphoid tissue (GALT) to initiate and regulate the host innate and adaptive immune responses. For example, Lactobacillus and Bifidobacterium species can induce dendritic cells to produce the anti-inflammatory cytokine IL-10 and suppress the production of the pro-inflammatory cytokine IFN-γ, thereby attenuating intestinal inflammatory responses [23,24]. On the other hand, probiotics can also modulate the immune function of intestinal B cells, promoting the secretion of IgA and other antibodies and thereby strengthening the defensive capacity of the intestinal mucosal barrier [25,26]. In addition, the antioxidant properties of probiotics represent another important mechanism by which they alleviate intestinal stress. Relevant studies have shown that probiotics can modulate the activity of antioxidant enzymes, thereby reducing oxidative stress–induced damage to intestinal tissues and consequently decreasing oxidative injury and apoptosis in intestinal epithelial cells [5,27].

Despite the widely recognized beneficial effects of probiotics, the synergistic actions among different strains and their combinations, as well as the precise mechanisms by which they act in sheep, remain to be fully elucidated. Therefore, to determine whether the sheep-derived probiotic strains previously isolated in our laboratory can alleviate diarrhea by improving intestinal health, we conducted a study in early-weaned lambs. Specifically, we systematically evaluated the regulatory effects of a sheep-derived probiotic mixture on intestinal immune function, barrier integrity, and antioxidant capacity, and further assessed its efficacy in reducing the incidence of diarrhea and promoting growth and development in lambs.

## 2. Materials and Methods

### 2.1. Animal Ethical Statement

This experiment was approved by the Animal Ethics Committee of Shihezi University (approval date: 5 June 2024; approval code: A2024-428). The experiment was conducted at Shangpin Meiyang Co., Ltd., Changji, China.

### 2.2. Animals and Diets

In this experiment, 60 healthy newborn Hu sheep lambs (1 d of age) were enrolled. All lambs were dam-suckled and had ad libitum access to pelleted feed and water. Lambs were randomly allocated to three groups. Lambs in the control group (CON) were continuously suckled until 35 d of age. The remaining two groups were weaned at 30 d of age and reared until 35 d of age, when lambs were humanely euthanized for sample collection. Among the weaned lambs, those that did not receive any supplements were assigned to the early-weaning group (EW). Lambs in the early-weaning plus probiotic group (EWP) received daily oral supplementation with *Lactiplantibacillus plantarum* M1 and *Limosilactobacillus reuteri* K4, both isolated from sheep in our laboratory, at a concentration of 1 × 10^9^ CFU/g and an administration amount of 2 g/day (i.e., 2 × 10^9^ CFU per lamb per day) from 1 day of age until weaning. Multistrain probiotics are increasingly adopted in commercial livestock production systems owing to their enhanced functional stability and synergistic benefits compared with single-strain formulations, we evaluated a sheep-derived mixed formulation (*L. plantarum* M1 + *L. reuteri* K4) as a practical intervention against early-weaning stress. All lambs had ad libitum access to creep feed from 7 d of age. The ingredient composition and nutrient levels of the diet are shown in Table S1.

During the experimental period, diarrhea was monitored daily, and diarrhea scores and incidence were calculated (Table S2) [28]. A daily score of ≥3 was classified as diarrhea according to a standardized protocol. Feed intake was recorded daily, and lambs were weighed once every two weeks before the morning feeding to calculate growth performance. The incidence of diarrhea in lambs was calculated as follows: incidence of diarrhea (%) = (number of diarrheic lambs in each group × days with diarrhea)/(total number of lambs in each group × experimental days) × 100.

### 2.3. Sample Collection

On day 35 of the experiment, jugular blood was collected from six lambs in each group before the morning feeding using a disposable blood collection needle and two 10 mL heparinized gel vacuum tubes. After resting for 20 min, the blood samples were centrifuged for 15 min at 3000× *g* at 4 °C, and the supernatant was collected. The supernatant was aliquoted into 2 mL centrifuge tubes and stored in liquid nitrogen for subsequent determination of biochemical, antioxidant, and immunological parameters.

On the last day of the experiment, six lambs from each group were randomly selected and transferred to a slaughterhouse for euthanasia. Immediately after slaughter, the intestinal tract and visceral organs were removed. Subsequently, segments of the jejunum, ileum, and colon were rapidly placed in 4% paraformaldehyde solution and fixed. Another portion of colonic tissue was placed in 2 mL cryovials and immediately stored in liquid nitrogen for transcriptomic analysis.

### 2.4. Biochemical Assays

Colonic tissues were homogenized in pre-cooled saline to obtain 10% (*w*/*v*) tissue homogenates, which were then centrifuged, and the supernatants were collected. Plasma levels of glucose (GLU), total protein (TP), albumin (ALB), globulin (GLB), total bilirubin (TBIL), direct bilirubin (DBIL), indirect bilirubin (IBIL), triglycerides (TG), total cholesterol (TC), low-density lipoprotein cholesterol (LDL), and high-density lipoprotein cholesterol (HDL) were measured using a fully automated biochemical analyzer. Colonic and plasma activities of superoxide dismutase (SOD), catalase (CAT), glutathione peroxidase (GSH-Px), and total antioxidant capacity (T-AOC), as well as malondialdehyde (MDA) concentrations, were determined using commercial biochemical reagent kits (Jiancheng, Nanjing, China). Colonic and plasma concentrations of interleukin-1β (IL-1β), tumor necrosis factor-α (TNF-α), interleukin-10 (IL-10), transforming growth factor-β (TGF-β), immunoglobulin A (IgA), immunoglobulin G (IgG), and immunoglobulin M (IgM), as well as colonic secretory IgA (sIgA), were quantified using ELISA kits (Jiancheng, Nanjing, China). Colonic levels of mucin 2 (MUC2), occludin, claudin-1, zonula occludens-1 (ZO-1), lipopolysaccharide (LPS) and diamine oxidase (DAO) were also quantified using ELISA kits (Jiancheng, Nanjing, China).

### 2.5. Histological Analysis

The tissues were stored in 4% paraformaldehyde for 24 h, followed by dehydration through graded ethanol. Tissue sections (5 μm thick) were stained with hematoxylin and eosin (H&E) and examined under a microscope (Olympus, Tokyo, Japan). At the same time, the depth of crypts in colon tissue and the average number of goblet cells per colon crypt, as well as the villus height and crypt depth in the duodenum and jejunum, were measured using ImageJ v1.8.0 software (National Institutes of Health, Bethesda, MD, USA).

### 2.6. Transcriptomic Analysis of Colon

Total RNA was extracted from colonic tissue using TRIzol reagent (Takara Bio, Otsu, Japan). RNA integrity was assessed using a 5300 Bioanalyzer (Agilent Technologies, Santa Clara, CA, USA), and RNA concentration was quantified using a NanoDrop spectrophotometer (Thermo Fisher Scientific, Waltham, MA, USA). High-quality RNA was used to construct sequencing libraries, which were sequenced on the DNBSEQ-T7 platform (PE150) at Shanghai Majorbio Biopharm Biotechnology Co., Ltd. (Shanghai, China). Differentially expressed genes (DEGs) were identified using DESeq2. Genes with |fold change| > 2 and Benjamini–Hochberg FDR-adjusted *p*-values (padj) < 0.05 were considered differentially expressed. Gene Ontology (GO) enrichment analysis of DEGs was performed using topGO (Gene Ontology Consortium, http://geneontology.org/). The Kyoto Encyclopedia of Genes and Genomes (KEGG) database was used to analyze DEGs involved in major biochemical metabolic and signaling pathways (https://www.genome.jp/kegg/, accessed on 10 December 2025). Weighted gene co-expression network analysis (WGCNA) was used to characterize gene association patterns across samples and to identify co-expressed gene modules, as well as to explore associations between gene modules and phenotypes of interest, and hub genes within each module (https://horvath.genetics.ucla.edu/html/CoexpressionNetwork/Rpackages/WGCNA/faq.html, accessed on 10 December 2025). Gene set enrichment analysis (GSEA) of hub genes (grouped according to median expression level) was performed using the clusterProfiler package (v4.18.4). Hub genes were further prioritized using the MCC algorithm implemented in the CytoHubba plugin (v0.1).

### 2.7. Validation of RNA-Seq

Total RNA from colonic tissue was extracted using a TRIzol reagent kit (TransGen Biotech Co., Ltd., Beijing, China), quantified with a NanoDrop spectrophotometer (Thermo Fisher Scientific Waltham, MA, USA), and reverse-transcribed into cDNA using a cDNA synthesis kit (Vazyme, Nanjing, China). The resulting cDNA was stored at −20 °C until further analysis. Quantitative real-time PCR (qPCR) was then performed on a Roche LightCycler 96 system (Roche, Mannheim, Germany) using 1 µL of a 1:20 diluted cDNA sample as the template for each reaction. Relative gene expression was calculated using the 2^−ΔΔCt^ method, with β-actin serving as the internal reference gene. Primer sequences are listed in Supplementary Table S3.

### 2.8. Statistics and Analysis

All experimental data were first entered into Microsoft Excel 2024 for organization and then imported into SPSS 27.0 Statistics (IBM Corp., Chicago, IL, USA) for analysis. Data on lamb body weight, feed intake, fecal score, and incidence of diarrhea were analyzed using a general linear model, with the effect of Hu sheep specified as a random effect and diet and time specified as fixed effects. The diet × time interaction was also included in the model. For parameters related to intestinal tissue samples, mRNA expression, plasma biochemical indices, and antioxidant capacity, the individual sheep was considered the experimental unit. These variables were analyzed by one-way ANOVA, and differences among means were assessed using the least significant difference (LSD) test in SPSS Statistics for Windows (version 19.0; IBM Corp., Chicago, IL, USA). All data are expressed as means ± SEMs. Differences were considered statistically significant at *p* < 0.05.

## 3. Results

### 3.1. Growth and Diarrhea of Lambs

Growth performance (Table 1) showed that although BW and ADG at individual time points did not differ among treatments (*p* > 0.05), and no overall treatment effect was detected for BW (*p* = 0.561) or ADG (*p* = 0.184), BW and ADG increased over time (time effect, *p* < 0.001). EW lambs had higher DMI (*p* < 0.001) and lower feed efficiency (*p* = 0.001) during days 31–35. In contrast, probiotic supplementation partially improved this parameter. Fecal scores were not affected by treatment (*p* > 0.05), but diarrhea incidence increased in EW relative to CON, whereas EWP showed a lower incidence than EW (Table 2). Overall, early weaning impaired growth and feed efficiency and increased diarrhea risk, while mixed probiotics exerted a partial protective effect.

### 3.2. Blood Biochemical Measurements

Plasma GLU and ALB were not affected by treatment (*p* > 0.05, Table 3). In contrast, EW reduced TP and GLB compared with the CON (*p* < 0.05), while EWP showed intermediate values. Early weaning also altered lipid metabolism, as TG, TC, and HDL concentrations were lower in EW and EWP groups than in CON (*p* < 0.05), whereas LDL only tended to decrease without statistical significance (*p* > 0.05). Liver function markers were largely unchanged, except for a reduction in DBIL in EW compared with CON (*p* < 0.05).

### 3.3. Blood Antioxidant Index, Inflammatory Cytokines and Immune Indicators

Probiotic supplementation markedly modulated plasma antioxidant and immune indices in early-weaned lambs. EW decreased CAT, GSH-PX and SOD activities and increased MDA concentrations compared with the CON (*p* < 0.001). Probiotic supplementation restored CAT and GSH-PX and normalized MDA toward CON values; however, plasma SOD activity in EWP remained significantly lower than CON, with no significant differences in T-AOC among groups (*p* = 0.103, Table 4). EW also elevated the pro-inflammatory cytokines IL-1β and TNF-α (*p* < 0.001) and reduced the anti-inflammatory cytokines IL-10 and TGF-β (*p* < 0.001), whereas EWP partially reversed these alterations. In addition, EW increased plasma IgA and IgM and yielded the highest IgG concentrations (*p* < 0.001), while probiotic supplementation shifted immunoglobulin profiles toward those observed in CON lambs (Table 5).

### 3.4. Effects of Feeding Mixed Probiotics on Intestinal Morphology in Lambs

**Figure 1:**
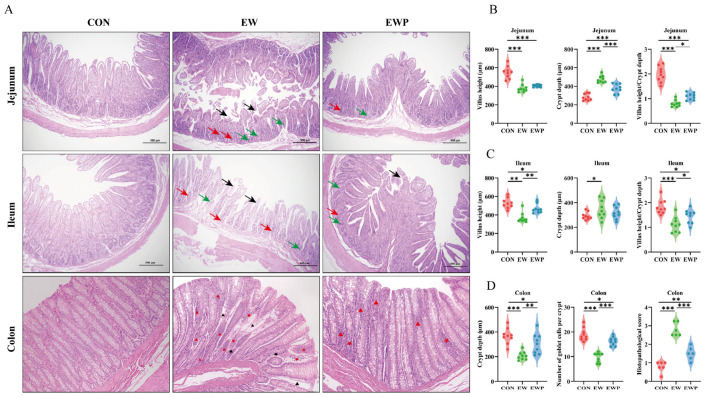
The protective effect of mixed probiotics on intestinal damage caused by weaning. (**A**) Representative H&E staining of jejunal, ileum and colon tissue across three treatment groups: CON, EW, and EWP. Black arrows: villus atrophy/shortened villi; red arrows: inflammatory cell infiltration; green arrows: crypt atrophy or loss of crypt structure (Scale bars. 500 μm), shown in jejunal and ileal tissues. Black star: cryptal inflammation; red star: crypt abscess; black triangle: loss of crypt structure; red triangle: goblet cell loss (Scale bars, 50 μm), shown in colonic tissue. Jejunal (**B**) and Ileum (**C**) villus height, crypt length, ratio of the villus height and crypt length. (**D**) Colonic crypt depth, number of goblet cells per crypt, and histopathological score. Data are presented as means ± SEM (*n* = 6). * *p* < 0.05, ** *p* < 0.01, *** *p* < 0.001.

EW markedly impaired intestinal morphology and barrier function. In the jejunum and ileum, EW reduced villus height and the villus height-to-crypt depth (VH/CD) ratio, increased crypt depth (*p* < 0.05 or *p* < 0.001), and induced villus blunting, epithelial shedding, and inflammatory cell infiltration (Figure 1A). EWP decreased crypt depth, increased the VH/CD ratio (*p* < 0.001), and partially restored mucosal architecture, alleviating tissue injury. In the colon, EW increased crypt depth, reduced goblet cell numbers, and elevated histopathological scores (*p* < 0.01), whereas EWP mitigated these changes (Figure 1B). Consistently, colonic Muc2, ZO-1, claudin-1, and occludin contents were reduced in EW lambs (*p* < 0.05), and probiotic supplementation partially reversed these decreases (Table 6).

### 3.5. Colon Mucosal Antioxidant Index, Inflammatory Cytokines and Immune Indicators

Early weaning markedly impaired intestinal antioxidant status (Table 7), as reflected by decreased CAT, T-AOC, GSH-PX, and SOD activities and increased MDA concentrations compared with the CON (*p* < 0.001), whereas EWP partially restored antioxidant enzyme activities and normalized MDA to control levels. EW also elevated intestinal IL-1β and TNF-α and reduced IL-10 and TGF-β (*p* < 0.001), with EWP significantly attenuating these inflammatory alterations (Table 8). Moreover, EW reduced DAO and increased sIgA, LPS, and mucosal IgA, IgM, and IgG (*p* < 0.001), while EWP generally shifted these indices toward CON values, particularly for IgA and IgG, indicating that early weaning compromises intestinal antioxidant defenses and mucosal immunity, whereas probiotic supplementation confers partial protection.

### 3.6. Probiotics Regulates the Colon Transcriptome Against Weaning-Induced Colitis in Lambs

**Figure 2:**
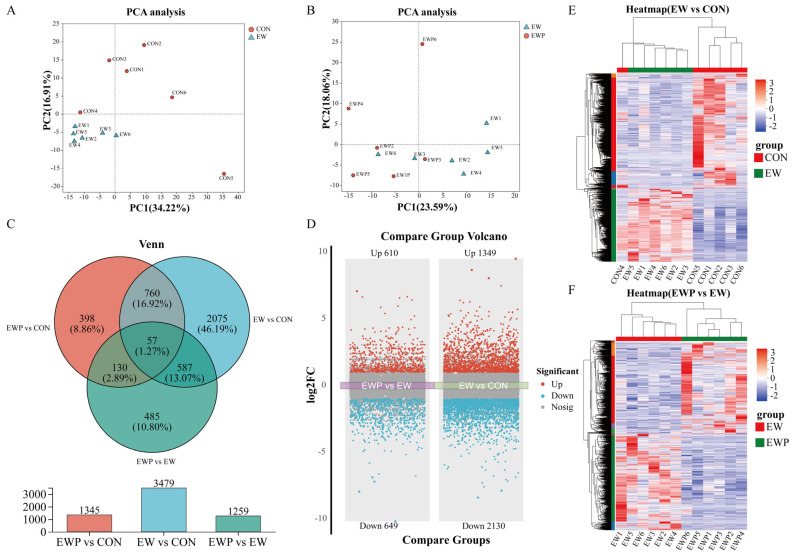
Transcriptomic analysis of colon mucosa. (**A**) Principal component analysis (PCA) of gene expression profiles comparing CON and EW groups. (**B**) PCA plot comparing EW and EWP groups. (**C**) Venn diagram showing overlap of differentially expressed genes (DEGs) among three comparisons. (**D**) Volcano plots showing DEG distribution. Left panel: EWP vs. EW comparison. Right panel: EW vs. CON comparison. (**E**) Hierarchical clustering heatmap of DEGs between EW and CON groups. (**F**) Hierarchical clustering heatmap of DEGs between EWP and EW groups (*n* = 6). The red and green colors represent the positions of the samples. The colors on the left side of the graph indicate different clusters of genes.

To further confirm the potential mechanism of mixed probiotics in alleviating weaning-induced intestinal inflammation in lambs, we conducted RNA sequencing on colon tissue. Principal component analysis (PCA) showed clear separation among the CON, EW, and EWP groups, indicating distinct transcriptomic profiles associated with early weaning and probiotic supplementation (Figure 2A,B). The Venn diagram revealed that 3479 differentially expressed genes (DEGs) were identified between EW and CON groups, while 1345 and 1259 DEGs were detected in EWP vs. CON and EWP vs. EW comparisons (Figure 2C). Specifically, Volcano plots demonstrated that early weaning induced substantial gene expression changes, with 1349 upregulated and 2130 downregulated genes compared to the CON group. Probiotic supplementation partly modulated these changes, showing 610 upregulated and 649 downregulated genes relative to the EW group (Figure 2D). Hierarchical clustering analysis further confirmed distinct gene expression patterns between the EW and CON groups, as well as between the EWP and EW groups (Figure 2E,F). These results indicate that early weaning markedly alters colonic gene expression, and probiotic supplementation can partially mitigate these effects.

**Figure 3:**
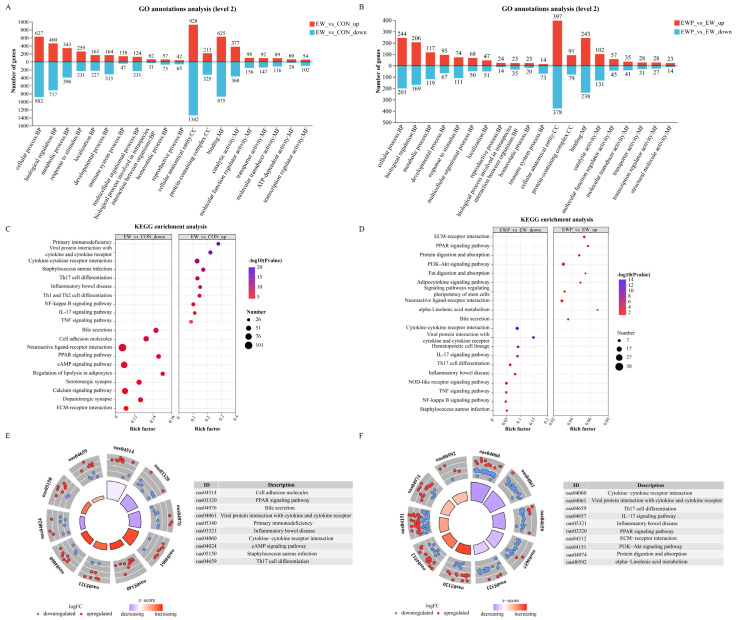
GO and KEGG pathway enrichment analysis of differentially expressed genes. (**A**) GO and (**C**) KEGG enrichment analysis of DEGs in the EW vs. CON. (**B**) GO and (**D**) KEGG enrichment analysis of DEGs in the EWP vs. EW. (**E**) Circos diagram of the KEGG pathways enrichment analysis in the EW vs. CON. (**F**) Circos diagram of the KEGG pathways enrichment analysis in the EWP vs. EW. The up-regulated and down-regulated genes in the figure are represented by red and blue colors, respectively. Among them, the change from purple to red in the inner circle represents the z-score value, which is used to estimate whether the biological process may be activated or inhibited. The first column in the table is the KEGG ID shown in the figure, and the second column is the corresponding description information.

Gene ontology (GO) enrichment analysis of these differentially expressed genes indicated that the EW group exhibited upregulation in key biological processes such as the immune system process (GO: 0002376) and response to stimulus (GO:0050896), downregulation in key biological processes such as cellular process (GO:0009987), metabolic process (GO:0008152) and developmental process (GO:0032502) (Figure 3A). In addition, Kyoto Encyclopedia of Genes and Genomes (KEGG) enrichment showed that weaning significantly affected immune-related pathways including Cytokine-cytokine receptor interaction, Th17 cell differentiation, Staphylococcus aureus infection, and Inflammatory bowel disease signaling (Figure 3C), suggesting an enhanced pro-inflammatory response. In parallel, several metabolic and developmental processes were downregulated, indicating immune and metabolic dysregulation during weaning stress. GO enrichment analysis of the EWP group exhibited upregulation of cellular process (GO:0009987) and developmental process (GO:0032502), downregulation in key biological processes such as immune system process (GO: 0002376) and response to stimulus (GO:0050896) (Figure 3B). KEGG enrichment showed that EWP influenced pathways related to PPAR signaling, lipid metabolism, and inflammation regulation (Figure 3D). Representative pathway gene expression profiles (Figure 3E,F) showed up- or down-regulation of key immune and metabolic genes. The evidence indicating that probiotic supplementation alleviates weaning-induced inflammatory and metabolic disturbances.

### 3.7. Weighted Correlation Network Analysis

**Figure 4:**
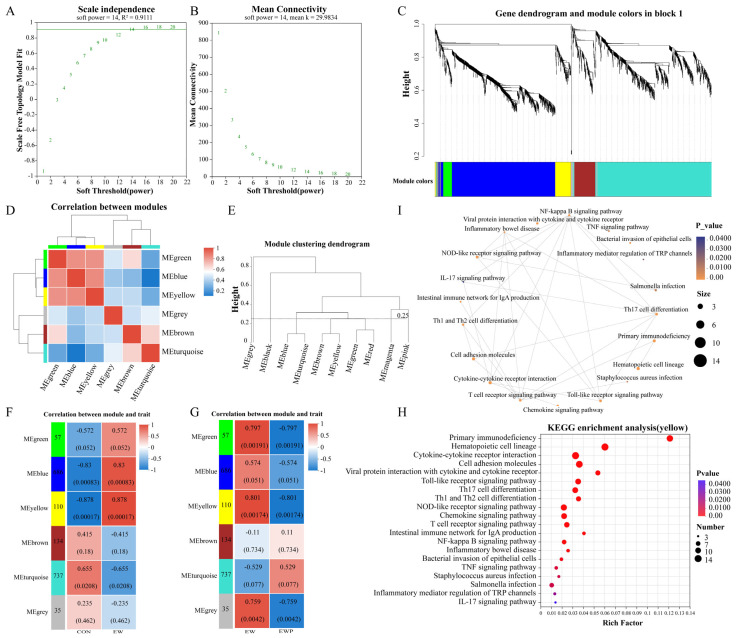
Weighted gene co-expression network analysis (WGCNA) identifies key modules associated with weaning stress and probiotic intervention. (**A**) Scale-free topology fit index as a function of soft-thresholding power (β). (**B**) Mean connectivity as a function of β, with β = 14 selected. (**C**) Gene dendrogram and module color assignment showing 6 distinct co-expression modules. (**D**) Heatmap of module-module correlations based on eigengene values. (**E**) Hierarchical clustering dendrogram of module eigengenes. (**F**) Module-trait correlation heatmap for early weaning (EW) group and CON, with correlation coefficients and *p*-values shown. (**G**) Module-trait correlation heatmap for probiotic (EWP) group and early weaning (EW) group. (**H**) KEGG pathway enrichment bubble plot showing significantly enriched pathways MEyellow modules, with bubble size representing gene number and color indicating *p*-value. (**I**) The enrichment analysis network diagram of the MEyellow module shows the interaction relationships among the enriched pathways.

To systematically identify gene modules associated with weaning stress and probiotic intervention, we performed WGCNA on the transcriptome data. A soft-thresholding power of β = 14 was selected based on scale-free topology criteria (R^2^ = 0.911) that achieved scale-free topology while maintaining high mean connectivity (k = 29.9834), ensuring biologically meaningful module detection (Figure 4A,B). Using dynamic tree cutting with a minimum module size of 30 genes and a merge cut height of 0.25, we identified 6 distinct co-expression modules (Figure 4C). Module correlation analysis (Figure 4D) revealed that MEgreen, MEblue, and MEyellow showed strong positive inter-module correlations, suggesting functional coordination, while MEturquoise exhibited negative correlations with these modules, indicating opposing regulatory patterns. The module clustering dendrogram (Figure 4E) further revealed the hierarchical relationships among modules. This clustering pattern suggests that modules within the same cluster may participate in related biological processes or respond coordinately to experimental treatments. To identify modules significantly associated with experimental groups (CON, EW, EWP), we correlated module eigengenes with sample traits (Figure 4F,G). MEyellow modules were strongly positively correlated with weaning stress (r = 0.878, *p* = 0.00017), but negatively correlated with probiotic intervention (r = −0.801, *p* = 0.00174). KEGG enrichment analysis and network of MEyellow, the most weaning-responsive module, revealed significant enrichment in inflammatory and immune pathways, including NF-kappa B signaling, cytokine-cytokine receptor interaction, NOD-like receptor signaling, Toll-like receptor signaling, IL-17 signaling pathway, Th1/Th2 and Th17 cell differentiation, inflammatory bowel disease, and pathogen infection pathways (Salmonella infection, Staphylococcus aureus infection) (Figure 4H,I).

### 3.8. GSEA and Hub Gene Analysis of MEyellow Module

**Figure 5:**
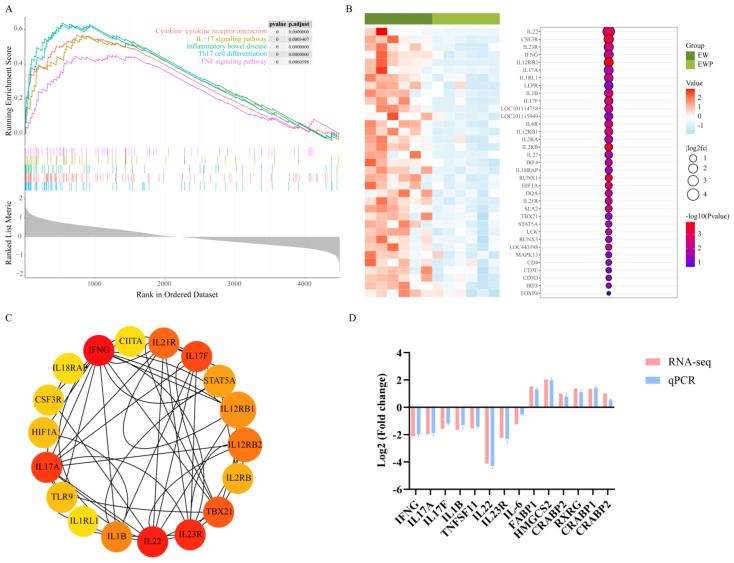
GSEA and hub gene analysis of MEyellow module. (**A**) GSEA enrichment plots showing inflammatory pathways enriched in EW vs. EWP groups. The pathways shown include Cytokine-cytokine receptor interaction (red), IL-17 signaling pathway (yellow-green), Inflammatory bowel disease (green), Th17 cell differentiation (blue), and TNF signaling pathway (purple). The gray shaded area represents the Ranked List Metric, indicating the baseline measure of variation in the dataset. The horizontal axis represents the positions of all genes, while the vertical axis represents the degree of association between the gene and the phenotype. Statistical significance is shown for each pathway with *p*-values and adjusted *p*-values displayed in the upper-right corner. (**B**) Heatmap of Th17 cell differentiation hub gene expression (left) and statistical significance (right) (*n* = 6). Bubble size represents log2 (fold change); color intensity indicates -log10 (*p*-value). (**C**) PPI network of hub genes. Node size and color represent connectivity degree. (**D**) qPCR validation of selected DGEs genes (*n* = 3). Pink bars: RNA-seq; blue bars: qPCR.

Gene Set Enrichment Analysis (GSEA) was performed to identify the biological functions of MEyellow module genes. As shown in Figure 5A, genes in this module were significantly enriched in pro-inflammatory pathways in the EW group, including Cytokine-cytokine receptor interaction, IL-17 signaling pathway, Inflammatory bowel disease, Th17 cell differentiation, and TNF signaling pathway (all |NES| > 2.0, *p* < 0.001). The enrichment curves demonstrated that these inflammatory pathways were activated by weaning stress but markedly suppressed in the EWP group. Specifically, the core genes in the Th17 signaling pathway, including pro-inflammatory cytokines (*IL22*, *IL23R*, *IL17A*, *IL1B*, *IL17F*), chemokines (*CSF3R*), transcription factors (*IFNG*, *TBX21*, *STAT5A*), and immune receptors (*IL12RB1*, *IL12RB2*, *IL18RAP*, *TLR6*) showed marked downregulation following probiotic intervention (Figure 5B). The PPI network illustrated the complex interactions among hub genes, with key inflammatory mediators such as *IFNG*, *IL17A*, *IL1B*, *IL22,* and *TBX21* serving as central nodes with high connectivity, indicating their critical roles in the weaning stress-induced inflammatory response (Figure 5C). We randomly selected eight hub genes and five PPAR pathway DEGs to verify the reliability of the sequencing results. The results showed that the expression patterns of these genes in qPCR and RNA-seq were consistent (Figure 5D). These results indicate that the MEyellow module represents a core inflammatory network activated by early weaning stress, and probiotic supplementation effectively suppresses this inflammatory response by downregulating key hub genes involved in cytokine signaling and immune regulation.

## 4. Discussion

In early life, lambs rely on passive immunity acquired from colostrum. When this passive transfer is inadequate, their resistance to environmental pathogens is reduced, predisposing them to diarrhea. In addition, changes in diet composition and separation from the dam after weaning further increase diarrhea risk in lambs [29,30]. Diarrhea impairs the digestion and absorption of nutrients, leading to growth retardation and thereby compromising subsequent growth and reproductive performance [31,32]. Although antibiotic therapy is currently one of the most effective strategies for controlling diarrhea, antibiotic residues and the emergence of antimicrobial resistance pose serious threats to animal and public health. Therefore, there is an urgent need to develop safe, “green” microbial alternatives to antibiotics that can reduce the incidence of diarrhea and promote intestinal health, which has become a major goal of modern animal husbandry [33]. Beyond probiotics, other nutritional strategies, such as liquid whey supplements, have been reported to modulate intestinal histology and immune-inflammatory responses during weaning stress in pigs [34,35], suggesting that targeted nutrition can enhance gut mucosal defense during the early life transition. We acknowledge that the absence of single-strain groups limits strain-specific attribution; future studies will compare single strains and their combination to quantify additive versus synergistic effects. In this context, we investigated the preventive effect of dietary supplementation with a mixture of the probiotic strains *L. plantarum* M1 and *L. reuteri* K4 on weaning-induced diarrhea in lambs. Furthermore, by combining analyses of intestinal morphology with transcriptomic profiling, we explored the mechanisms underlying the interaction between intestinal health and systemic immunity.

Building on this background, previous studies have shown that weaning stress reduces feed intake and increases the incidence of diarrhea in lambs [36], thereby restricting their growth and development [37,38]. Consistent with these reports, our results showed that weaning significantly reduced feed efficiency and increased diarrhea incidence, leading to decreases in both body weight and average daily gain in weaned lambs [39]. Probiotic supplementation reduced the incidence of diarrhea to a level comparable to that observed in continuously suckled control lambs. Weaning also induced pronounced metabolic and immune alterations. Early weaning significantly decreased serum TP and GLB concentrations, indicating impaired protein synthesis and/or increased protein catabolism [40,41]. In addition, lipid metabolism markers, TG, TC, and HDL, were simultaneously reduced, consistent with the concept that weaning stress activates the hypothalamic–pituitary–adrenal axis and promotes protein and fat mobilization to meet increased energy demands [42,43].

In line with these systemic changes, cytokines with pro-inflammatory and anti-inflammatory properties are key components of cell-mediated immunity [44]. In the present study, plasma IL-1β and TNF-α levels were elevated, whereas IL-10 and TGF-β levels were reduced, indicating that early weaning induced a systemic pro-inflammatory state. The increased concentrations of immunoglobulins (IgA, IgM, IgG) in early-weaned lambs likely reflect compensatory immune activation in response to enhanced antigen exposure caused by impaired intestinal barrier function [40,45]. Supplementation with the mixed probiotics partially reversed the elevations in pro-inflammatory cytokines and immunoglobulins. Weaning stress is also known to generate excessive free radicals and trigger oxidative stress [30]. In this study, we showed that dietary supplementation with the mixed probiotics conferred marked antioxidant benefits, as evidenced by a significant reduction in plasma MDA, a lipid peroxidation product, and increased activities of the antioxidant enzymes CAT and GSH-Px. Collectively, these findings suggest that the probiotics may alleviate weaning-induced growth retardation and increased diarrhea by modulating intestinal inflammatory signaling, enhancing intestinal barrier integrity, and attenuating systemic oxidative stress.

At the intestinal structural level, damage to intestinal morphology severely impairs the digestion and absorption of nutrients [46]. The intestinal barrier maintains gut health by preventing pathogens, toxins, and antigens from crossing the intestinal mucosa into the bloodstream [47]. Intestinal histomorphology has been widely used to assess the development and renewal of intestinal epithelial cells [48]. In general, increased villus height enlarges the absorptive surface area of the small intestine, whereas reduced crypt depth and a higher villus height-to-crypt depth ratio (VH/CD) indicate slower epithelial turnover and more mature enterocytes, which are associated with improved nutrient absorption and growth performance [49,50]. Our results showed pronounced villus atrophy and a reduced villus height-to-crypt depth ratio in the jejunum and ileum of weaned lambs, corroborating the decreases in feed efficiency and body weight after weaning. Previous studies have demonstrated that supplementation with *L. reuteri* L81 and *L. johnsonii* M5 significantly increases villus height, decreases crypt depth, and consequently increases the villus height-to-crypt depth ratio in calves and lambs [27,51], which is consistent with our findings. Tight junctions, which are composed of transmembrane proteins such as claudins and occludins together with scaffold proteins (e.g., ZO family proteins), are responsible for maintaining the integrity of the intestinal barrier and regulating intestinal permeability [52]. In addition, MUC2 secreted by goblet cells is the major structural component of the colonic mucus layer and is crucial for maintaining epithelial barrier integrity, blocking bacteria and toxins, and preventing colitis [53]. Supplementation with mixed probiotics attenuated the weaning-induced reduction in colonic goblet cell numbers and the increase in histopathological scores, and significantly upregulated the levels of tight junction proteins and MUC2. Consistent with previous findings, supplementation with sheep-derived *C. beijerinckii* R8 and bovine-derived *L. reuteri* L81 markedly increased both the protein abundance and gene expression of tight junction components in the colon and jejunum [2,51].

Diarrhea is commonly accompanied by intestinal inflammation and redox imbalance within the gut [54,55]. There is evidence that the hindgut microbiota of early-weaned goats promotes the expression of inflammatory cytokines [12], and that disturbed intestinal redox homeostasis further stimulates immune responses [54]. Probiotics can modulate cytokine expression and reduce inflammation, thereby activating and shaping host immune responses [56]. In accordance with previous studies, supplementation with mixed probiotics reduced the levels of the pro-inflammatory cytokines IL-1β and TNF-α, while increasing the levels of the anti-inflammatory mediators IL-10 and TGF-β [27,57]. In weaned lambs, elevated intestinal LPS concentrations together with reduced DAO levels indicate impaired intestinal barrier integrity [30,58]. Damage to both the epithelial and mucus barriers increases the luminal antigen load and facilitates the translocation of bacteria and LPS into the mucus layer, which excessively stimulates B-cell activation and antibody production [59,60], leading to increased concentrations of sIgA, IgA, IgM, and IgG in plasma and mucosa. Probiotic supplementation improved intestinal morphology and tight junction protein expression, attenuated local and systemic inflammation, and consequently reduced antigenic pressure, thereby restoring mucosal immunoglobulin levels toward homeostatic values [61]. Weaning-induced oxidative stress may arise from excessive production of reactive oxygen species (ROS) by inflammatory cells [62], mitochondrial dysfunction [63], and insufficient dietary antioxidants [64]. In our experiment, probiotic supplementation effectively enhanced intestinal antioxidant enzyme activities and reduced lipid peroxidation, indicating that mixed probiotics stimulate endogenous antioxidant defenses or directly scavenge ROS through bacterial antioxidant enzymes [65].

Transcriptomic analysis provides molecular-level insights into weaning-induced intestinal dysfunction and the mechanisms underlying probiotic-mediated protection. The study by Zhang et al. demonstrated that early weaning modulates host intestinal immune responses by altering the colonic epithelial transcriptional profile in lambs [12]. Consistent with these findings, our data showed that early weaning upregulated genes associated with immune system processes and inflammatory responses, while downregulating genes involved in cellular metabolism and developmental processes. KEGG pathway analysis further revealed significant enrichment of cytokine–cytokine receptor interaction, NF-κB signaling, TNF signaling, IL-17 signaling, Th1 and Th17 cell differentiation, and inflammatory bowel disease pathways. These findings indicate that early weaning activates both innate and adaptive immune pathways in the colonic mucosa, which is consistent with our phenotypic observations of elevated pro-inflammatory cytokines and immunoglobulins in the intestines of weaned lambs. Similar activation of NF-κB, TNF, and IL-17 related pathways has also been reported in the intestines of other weaning stress models and is considered a key driver of mucosal inflammation and barrier disruption [66,67]. Probiotic supplementation suppressed these excessively activated pathways. Previous studies have shown that lactic acid bacteria can downregulate NF-κB dependent inflammatory gene expression, reduce Th1/Th17 polarization, and promote a more balanced mucosal immune response in the immature intestine [68,69]. In addition to suppressing classical inflammatory pathways, probiotic supplementation markedly upregulated the PPAR signaling pathway. Activation of PPARα/γ not only coordinates lipid and energy metabolism but also exerts potent anti-inflammatory effects by inhibiting NF-κB and AP-1 driven transcription of inflammatory genes [70,71]. PPAR signaling was identified primarily at the transcript level. While these findings are consistent with PPAR-mediated anti-inflammatory regulation, we did not quantify PPAR proteins or phosphorylation states in the current cohort. Future work will include protein-level validation.

WGCNA identified several gene modules that were positively correlated with early weaning and negatively correlated with probiotic supplementation. The pathways enriched in these modules respond to increased antigenic and bacterial translocation, thereby enhancing the production of pro-inflammatory cytokines and driving a shift of the colon toward a Th1/Th17-type immune response. Inflammatory bowel disease (IBD) is characterized by excessive and uncontrolled immune responses mediated by Th1, Th2, or Th17 cells and their associated cytokines [72]. Hub genes within these modules, such as *IFNG*, are linked to Th1 cells in colitis, and Th1 cells promote cell-mediated inflammatory responses by inducing the activation of macrophages, NK cells, B cells, and CD8^+^ T cells [73]. Increased LPS levels in intestinal tissues engage membrane receptors, thereby enhancing NF-κB signaling and the secretion of the inflammatory mediator TNF-α, which in turn promotes the accumulation of immune cells in the tissue and causes tissue damage [74]. Consistent with previous clinical observations, patients with Crohn’s disease and ulcerative colitis exhibit marked accumulation of IL-17-producing Th17 cells and increased IL-23 expression in the intestinal mucosa [75]. Previous studies have also highlighted IFN-γ, IL-17A/F, and IL-22 as key effector cytokines that drive epithelial damage and increased permeability in IBD and infection models [76]. Probiotics have been shown to downregulate these cytokines and preserve epithelial integrity [77,78,79], which is consistent with our observations on intestinal barrier function. Our data support the notion that activation of the IL-23/Th17 axis is a central feature of intestinal inflammation. The downregulation of Th17-related genes by probiotic supplementation suggests that beneficial bacteria may modulate adaptive immune responses to prevent excessive inflammation.

Network-level analyses further support the concept that probiotics target key regulatory nodes controlling immune responses in the colon. PPI network analysis showed that hub genes such as *IFNG*, *IL17A*, *IL1B*, *IL22*, and *TBX21* occupy highly connected central positions, indicating that they play pivotal roles in coordinating inflammatory responses and represent potential therapeutic targets for alleviating weaning-induced intestinal inflammation. Previous studies have demonstrated that blocking receptors for Th17-associated pro-inflammatory cytokines can be used to treat IBD [80]. Consistent with our findings, *L. reuteri* ZY15 alleviates colitis by downregulating IL-17 and TNF-α [69]. In addition, oral administration of an engineered *Lactobacillus* strain that simultaneously targets IL-17A, IL-23, and TNF-α exerts synergistic effects in the treatment of IBD [81]. The marked upregulation of PPAR pathway components such as *FABP1*, *RXRG*, *CRABP1*, *CRABP2*, and *HMGCS2* suggests that probiotic intervention not only attenuates inflammatory responses but also remodels lipid and vitamin A metabolic programs in colonic epithelial cells. Vitamin A plays a key regulatory role in mucosal immunity by promoting the generation of Foxp3^+^ regulatory T cells and IgA production through retinoic acid signaling, while modulating dendritic cell function and guiding the differentiation and mucosal homing of effector T cells, thereby strengthening the mucosal barrier and immune protection [82]. Supplementation with vitamin-producing probiotic strains can also ameliorate IBD [83], further supporting the notion that probiotics promote restoration of mucosal homeostasis by improving the metabolic status of the epithelium.

**Figure 6:**
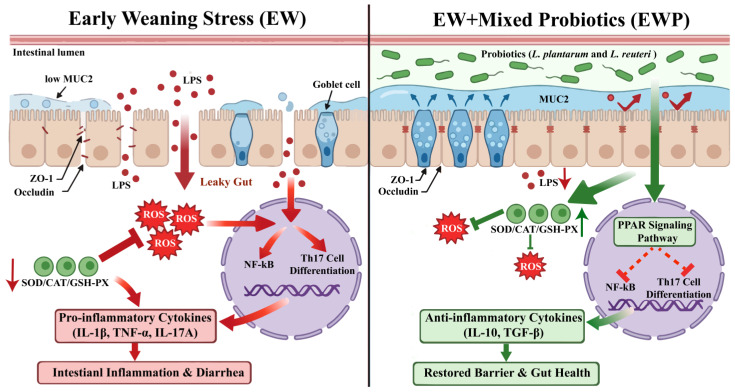
Schematic illustration of the protective effects of mixed probiotics on early weaning-induced intestinal injury. Under EW, MUC2 production is reduced and tight-junction proteins (ZO-1 and occludin) are disrupted, leading to increased intestinal permeability (“leaky gut”). LPS translocates across the epithelium and promotes oxidative stress (ROS accumulation), which activates NF-κB and facilitates Th17 cell differentiation, thereby elevating pro-inflammatory cytokines (IL-1β, TNF-α, and IL-17A) and resulting in intestinal inflammation and diarrhea. In contrast, EWP (*L. plantarum* and *L. reuteri*) enhances the mucus layer (MUC2) and tight-junction integrity, reduces LPS translocation, and boosts antioxidant defenses (SOD/CAT/GSH-PX). Activation of the PPAR signaling pathway may inhibit NF-κB activity and Th17 differentiation, increases anti-inflammatory cytokines (IL-10 and TGF-β), and ultimately restores barrier function and gut health. MUC2, mucin 2; ZO-1, zonula occludens-1; LPS, lipopolysaccharide; CAT, catalase; GSH-Px, glutathione peroxidase; SOD, superoxide dismutase; IL-1β, interleukin-1β; IL-10, interleukin-10; TNF-α, tumor necrosis factor-α; TGF-β, transforming growth factor-β; IL-17A, interleukin-17A.

In this addition, we elucidate the potential complementary mechanisms underlying the synergistic effects of *L. plantarum* M1 and *L. reuteri* K4. *L. plantarum* M1 contributes to antioxidant defense and barrier reinforcement likely through exopolysaccharide production and organic acid secretion, while *L. reuteri K4* enhances immune modulation and pathogen inhibition via reuterin production and biofilm formation. Multistrain formulations may by reinforcement of mucus/barrier integrity through microbial–epithelial interactions, and balanced immunomodulation that may reduce excessive Th1/Th17-skewed inflammation while supporting mucosal homeostasis. This was confirmed in our transcriptome data, and their combined effects may enhance the suppression of Th17-driven inflammation and activate the PPAR pathway. We compare these synergistic outcomes to single-strain studies, noting that while individual strains improve antioxidant status and reduce diarrhea [84,85], the combination in our study achieved broader benefits, such as more significant reductions in IL-1β, TNF-α, and MDA levels, restoration of SOD/GSH-Px activities and tight junction proteins. Our study demonstrates that mixed probiotic supplementation is a feasible nutritional strategy to mitigate weaning-associated intestinal dysfunction in lambs (Figure 6). Future work should employ in vitro cell-based models to elucidate the mechanisms by which probiotics regulate host gene expression and immune responses, include protein-level validation to confirm pathway activation and to link transcriptional changes to functional signaling outcomes.

## 5. Conclusions

In conclusion, this study demonstrates that supplementation with a mixed probiotic formulation effectively attenuated these weaning adverse effects by improving intestinal morphology, reducing markers of inflammation and oxidative stress, and modulating colonic gene expression to promote homeostasis. Transcriptomic analysis showed that probiotics suppressed weaning-induced inflammatory gene networks, particularly the Th17 cell differentiation pathway, while concurrently activating metabolic regulatory pathways such as PPAR signaling. Collectively, these findings provide molecular-level insights into the mechanisms underlying probiotic-mediated protection against weaning stress and support the use of probiotics as a nutritional strategy to improve intestinal health and production efficiency in early-weaned lambs.

## Figures and Tables

**Table 1:** Effects of mixed probiotics on growth performance of lambs.

Items	Times	Treatment ^1^			*p*-Value		
		CON	EW	EWP	T	D	T × D
BW, kg	1 d	4.89 ± 0.27	4.74 ± 0.21	4.72 ± 0.15	0.528		
	15 d	7.31 ± 0.53	7.36 ± 0.36	7.94 ± 0.27	0.410		
	30 d	12.21 ± 0.78	11.37 ± 0.49	11.78 ± 0.44	0.584		
	35 d	13.98 ± 0.78	12.98 ± 0.63	13.36 ± 0.47	0.153		
	Overall	9.94 ± 0.58	9.28 ± 0.40	9.52 ± 0.32	0.561	<0.001	0.013
ADG, g/d	1–30 d	245.48 ± 18.60	215.74 ± 11.78	235.37 ± 10.68	0.832		
	31–35 d	359.60 ± 25.93	322.92 ± 22.71	318.57 ± 10.73	0.307		
	Overall	262.57 ± 7.47	237.73 ± 11.33	244.38 ± 6.91	0.184	<0.001	0.718
DMI, g/d	1–30 d	75.97 ± 14.46	66.27 ± 12.88	54.68 ± 11.23	0.509		
	31–35 d	324.06 ± 2.64 ^c^	403.31 ± 8.64 ^a^	379.10 ± 9.19 ^b^	<0.001		
	Overall	111.41 ± 19.38	114.42 ± 23.26	101.02 ± 21.98	0.275	<0.001	<0.001
Feed efficiency, %	1–30 d	3.40 ± 0.24 ^b^	3.29 ± 0.18 ^b^	4.16 ± 0.20 ^a^	<0.001		
	31–35 d	1.11 ± 0.08 ^a^	0.79 ± 0.06 ^b^	0.85 ± 0.03 ^b^	0.001		
	Overall	2.36 ± 0.07 ^a^	2.08 ± 0.10 ^b^	2.41 ± 0.07 ^a^	<0.001	<0.001	<0.001

Note: BW, body weight; ADG, average daily gain; DMI, dry matter intake of starter feed; Feed efficiency, ADFI/ADG. ^1^ CON, ewe-reared group; EW, early weaning group; EWP, early weaning + mixed probiotics. Data are presented as the means ± standard errors of the means (SEMs) (*n* = 20). T, treatment; D, day; T × D, treatment × day. The same superscript letters indicate no significant difference (*p* > 0.05), while different letters indicate significant differences (*p* < 0.05).

**Table 2:** Effects of mixed probiotics on diarrhea rate and fecal score of lambs.

Items	Times	Treatment ^1^			*p*-Value		
		CON	EW	EWP	T	D	T × D
Fecal score	1–30 d	1.24 ± 0.06	1.26 ± 0.06	1.21 ± 0.05	0.402		
	31–35 d	1.22 ± 0.09	1.44 ± 0.09	1.38 ± 0.09	0.265		
	Overall	1.24 ± 0.06	1.30 ± 0.06	1.24 ± 0.05	0.337	0.006	0.316
Diarrhea incidence, %	1–30 d	7.08 ± 2.58	9.59 ± 2.65	4.84 ± 2.08	0.360		
	31–35 d	4.7 ± 2.12 ^c^	13.68 ± 3.44 ^a^	9.6 ± 2.61 ^b^	0.012		
	Overall	7.11 ± 2.50	10.51 ± 2.51	5.89 ± 2.03	0.123	0.004	0.412

Note: ^1^ CON, ewe-reared group; EW, early weaning group; EWP, early weaning + mixed probiotics. Data are presented as the means ± standard errors of the means (SEMs) (*n* = 20). T, treatment; D, day; T × D, treatment × day. The same superscript letters indicate no significant difference (*p* > 0.05), while different letters indicate significant differences (*p* < 0.05).

**Table 3:** Effect of mixed probiotics on plasma biochemical parameters of lambs.

Items	Treatment ^1^			*p*-Value
	CON	EW	EWP	
GLU, mmol/L	5.82 ± 0.32	5.84 ± 0.13	5.2 ± 0.16	0.104
TP, g/L	64.40 ± 1.84 ^a^	58.00 ± 0.78 ^b^	60.25 ± 1.45 ^ab^	0.020
ALB, g/L	31.35 ± 1.00	30.93 ± 0.52	31.85 ± 0.80	0.723
GLB, g/L	33.05 ± 1.89 ^a^	27.07 ± 1.05 ^b^	28.4 ± 1.22 ^b^	0.024
TG, mmol/L	0.53 ± 0.04 ^a^	0.39 ± 0.04 ^b^	0.35 ± 0.36 ^b^	0.015
TC, mmol/L	1.61 ± 0.16 ^a^	1.26 ± 0.05 ^b^	0.99 ± 0.10 ^b^	0.005
LDL, mmol/L	0.43 ± 0.05	0.39 ± 0.04	0.30 ± 0.05	0.203
HDL, mmol/L	1.24 ± 0.13 ^a^	0.90 ± 0.02 ^b^	0.75 ± 0.07 ^b^	0.003
TBIL, umol/L	2.55 ± 0.12	2.28 ± 0.07	2.28 ± 0.07	0.080
DBIL, umol/L	0.53 ± 0.05 ^a^	0.33 ± 0.06 ^b^	0.40 ± 0.05 ^ab^	0.047
IBIL, umol/L	2.02 ± 0.16	1.95 ± 0.07	1.88 ± 0.05	0.680

Note: GLU, glucose; TP, total protein, ALB, albumin; GLB, globulin; TBIL, total bilirubin; DBIL, direct bilirubin; IBIL, indirect bilirubin; TG, triglycerides; TC, total cholesterol; LDL, low-density lipoprotein cholesterol; HDL, high-density lipoprotein cholesterol. ^1^ CON, ewe-reared group; EW, early weaning group; EWP, early weaning + mixed probiotics. Data are presented as the means ± standard errors of the means (SEMs) (*n* = 6). The same superscript letters indicate no significant difference (*p* > 0.05), while different letters indicate significant differences (*p* < 0.05).

**Table 4:** Effects of mixed probiotics on plasma antioxidant index in lambs.

Items	Treatment ^1^			*p*-Value
	CON	EW	EWP	
CAT, U/mL	54.69 ± 2.02 ^a^	44.49 ± 1.51 ^b^	51.69 ± 1.92 ^a^	0.004
MDA, nmol/mL	0.53 ± 0.01 ^b^	0.74 ± 0.04 ^a^	0.60 ± 0.03 ^b^	<0.001
T-AOC, U/mL	3.56 ± 0.12	3.51 ± 0.04	3.79 ± 0.09	0.103
GSH-PX, U/mL	382.96 ± 14.08 ^a^	270.95 ± 13.11 ^b^	350.94 ± 14.74 ^a^	<0.001
SOD, U/mL	5.78 ± 0.30 ^a^	2.76 ± 0.47 ^b^	3.93 ± 0.38 ^b^	<0.001

Note: Plasma antioxidant indicators among different groups. CAT, catalase; MDA, malondialdehyde; T-AOC, total antioxidant capacity; GSH-Px, glutathione peroxidase; SOD, superoxide dismutase. ^1^ CON, ewe-reared group; EW, early weaning group; EWP, early weaning + mixed probiotics. Data are presented as the means ± standard errors of the means (SEMs) (*n* = 6). The same superscript letters indicate no significant difference (*p* > 0.05), while different letters indicate significant differences (*p* < 0.05).

**Table 5:** Effects of mixed probiotics on plasma inflammation cytokines and immune indicators in lambs.

Items	Treatment ^1^			*p*-Value
	CON	EW	EWP	
IL-1β, pg/mL	359.47 ± 12.64 ^c^	661.98 ± 13.65 ^a^	428.83 ± 8.22 ^b^	<0.001
IL-10, pg/mL	24.85 ± 0.45 ^a^	15.30 ± 0.40 ^c^	22.02 ± 0.53 ^b^	<0.001
TNF-α, pg/mL	181.45 ± 8.76 ^b^	257.87 ± 4.17 ^a^	254.95 ± 4.49 ^a^	<0.001
TGF-β, pg/mL	792.28 ± 14.73 ^a^	506.05 ± 4.03 ^c^	586.98 ± 16.53 ^b^	<0.001
IgA, μg/mL	576.90 ± 35.70 ^c^	972.85 ± 11.53 ^a^	737.34 ± 27.54 ^b^	<0.001
IgM, μg/mL	1346.83 ± 30.30 ^c^	2259.65 ± 53.92 ^a^	2001.93 ± 27.58 ^b^	<0.001
IgG, g/L	13.06 ± 0.71 ^b^	21.46 ± 0.54 ^a^	13.17 ± 0.73 ^b^	<0.001

Note: Plasma inflammation cytokines and immune indicators among different groups. IL-1β, interleukin-1β; IL-10, interleukin-10; TNF-α, tumor necrosis factor-α; TGF-β, transforming growth factor-β; IgA, immunoglobulin A; IgM, immunoglobulin M; IgG, immunoglobulin G. ^1^ CON, ewe-reared group; EW, early weaning group; EWP, early weaning + mixed probiotics. Data are presented as the means ± standard errors of the means (SEMs) (*n* = 6). The same superscript letters indicate no significant difference (*p* > 0.05), while different letters indicate significant differences (*p* < 0.05).

**Table 6:** Effects of mixed probiotics on colon barrier function-related protein in lambs.

Items	Treatment ^1^			*p*-Value
	CON	EW	EWP	
Muc2, ng/mg	1.52 ± 0.15 ^a^	0.86 ± 0.15 ^b^	1.23 ± 0.11 ^ab^	0.014
ZO-1, ng/mg	1.82 ± 0.03 ^a^	1.50 ± 0.03 ^c^	1.67 ± 0.02 ^b^	<0.001
Claudin-1, ng/mg	1.27 ± 0.19 ^a^	0.57 ± 0.14 ^b^	0.68 ± 0.13 ^b^	0.013
Occludin, ng/mg	2.33 ± 0.22 ^a^	1.72 ± 0.08 ^b^	2.12 ± 0.09 ^ab^	0.026

Note: MUC2, mucin 2; ZO-1, zonula occludens-1. ^1^ CON, ewe-reared group; EW, early weaning group; EWP, early weaning + mixed probiotics. Data are presented as the means ± standard errors of the means (SEMs) (*n* = 6). The same superscript letters indicate no significant difference (*p* > 0.05), while different letters indicate significant differences (*p* < 0.05).

**Table 7:** Effects of mixed probiotics oncolon antioxidant index in lambs.

Items	Treatment ^1^			*p*-Value
	CON	EW	EWP	
CAT, U/g	44.89 ± 1.75 ^a^	29.18 ± 2.07 ^b^	33.00 ± 2.36 ^b^	<0.001
MDA, nmol/g	0.81 ± 0.03 ^b^	1.27 ± 0.07 ^a^	0.89 ± 0.02 ^b^	<0.001
T-AOC, U/g	2.18 ± 0.07 ^a^	1.54 ± 0.14 ^b^	2.05 ± 0.08 ^a^	<0.001
GSH-PX, U/g	417.95 ± 21.87 ^a^	330.32 ± 21.30 ^b^	386.61 ± 17.83 ^ab^	0.026
SOD, U/g	34.17 ± 3.18 ^a^	18.16 ± 0.77 ^b^	24.56 ± 2.40 ^b^	<0.001

Note: Antioxidant indicators of the colon in different groups. CAT, catalase; MDA, malondialdehyde; T-AOC, total antioxidant capacity; GSH-Px, glutathione peroxidase; SOD, superoxide dismutase. ^1^ CON, ewe-reared group; EW, early weaning group; EWP, early weaning + mixed probiotics. Data are presented as the means ± standard errors of the means (SEMs) (*n* = 6). The same superscript letters indicate no significant difference (*p* > 0.05), while different letters indicate significant differences (*p* < 0.05).

**Table 8:** Effects of mixed probiotics oncolon inflammation cytokines and immune indicators.

Items	Treatment ^1^			*p*-Value
	CON	EW	EWP	
IL-1β, pg/mg	243.00 ± 10.68 ^c^	514.38 ± 21.71 ^a^	364.57 ± 9.14 ^b^	<0.001
IL-10, pg/mg	24.93 ± 0.50 ^a^	15.47 ± 0.53 ^c^	19.06 ± 0.55 ^b^	<0.001
TNF-α, pg/mg	166.65 ± 9.90 ^b^	294.2 ± 9.02 ^a^	178.25 ± 7.22 ^b^	<0.001
TGF-β, pg/mg	772.72 ± 19.62 ^a^	567.43 ± 22.12 ^c^	689.32 ± 23.29 ^b^	<0.001
LPS, EU/mL	374.30 ± 16.49 ^c^	734.50 ± 26.12 ^a^	522.32 ± 9.94 ^b^	<0.001
DAO, ng/mL	7.10 ± 0.11 ^a^	4.11 ± 0.29 ^c^	4.83 ± 0.25 ^b^	<0.001
sIgA, μg/mL	7.82 ± 0.52 ^c^	16.34 ± 0.41 ^a^	10.94 ± 0.67 ^b^	<0.001
IgA, μg/mL	422.72 ± 39.70 ^c^	947.32 ± 22.53 ^a^	648.50 ± 35.41 ^b^	<0.001
IgM, μg/mL	1489.63 ± 46.16 ^b^	2142.63 ± 49.03 ^a^	1496.17 ± 70.46 ^b^	<0.001
IgG, g/L	8.68 ± 0.49 ^b^	16.67 ± 0.47 ^a^	13.22 ± 0.90 ^c^	<0.001

Note: Inflammation cytokines and immune indicators indicators of the colon in different groups. IL-1β, interleukin-1β; IL-10, interleukin-10; TNF-α, tumor necrosis factor-α; TGF-β, transforming growth factor-β; LPS, lipopolysaccharide; DAO, diamine oxidase; sIgA, secretory immunoglobulin A; IgA, immunoglobulin A; IgM, immunoglobulin M; IgG, immunoglobulin G. ^1^ CON, ewe-reared group; EW, early weaning group; EWP, early weaning + mixed probiotics. Data are presented as the means ± standard errors of the means (SEMs) (*n* = 6). The same superscript letters indicate no significant difference (*p* > 0.05), while different letters indicate significant differences (*p* < 0.05).

## Data Availability

All original contributions presented in this study are included within the article, and further inquiries can be directed to the corresponding authors. The sequencing data generated in this study have been deposited in the Sequence Read Archive under the accession number: PRJNA1377242.

## Supplementary Materials

The following supporting information can be downloaded at: https://www.mdpi.com/article/10.3390/antiox15010132/s1, Table S1: Ingredient composition of basal diets (%, as-fed basis); Table S2: Fecal scoring system; Table S3: Primers for quantitative real time PCR.
