# Supplementary material for: Probiotic Combination of Lactiplantibacillus plantarum M1 and Limosilactobacillus reuteri K4 Alleviates Early Weaning-Induced Intestinal Injury in Lambs via Modulation of Oxidative and Inflammatory Pathways
Source: Antioxidants (Basel). 2026 Jan 20;15(1):132. doi: 10.3390/antiox15010132 (PMC12837899; doi:10.3390/antiox15010132)
Supplement: Supplementary file 1 [file antioxidants-15-00132-s001.zip › antioxidants-4065901-supplementary.pdf]

Supplemental materials

Table S1. Ingredient composition of basal diets (%, as-fed basis)[1]

Items	Content (%)
Ingredients	
Corn germ meal	39.0
Corn	26.0
Soybean meal	15.0
Wheat bran	10.0
Cottonseed meal	5.0
Limestone meal	2.3
Lurex	0.2
NaHCO3	1.0
NaCl	0.5
Premix[2]	1.0
Total	100
Nutrient content[3]	
OM	92.54
EE	2.43
CP	19.93
NDF	12.35
ADF	5.63
Ca	0.91
P	0.41

Note: [1]OM, Organic Matter; CP, crude protein; EE, ether extract; NDF, neutral detergent fiber; ADF, acid detergent fiber; Ca, calcium; P, phosphorus. [2]The premix provided the following per kg of the concentrate supplement: Cu 2.0 mg; Fe 40.0 mg; Zn 55.0 mg; Mn 65 mg; I 0.85 mg; Co (as cobalt chloride) 0.15 mg; Se (as sodium selenite) 0.05 mg; VA 8500 IU; VD 2080 IU; VE 25.00 mg; niacin 50.00 mg; biotin 0.15 mg. [3] Nutrient levels are all measured values.

Table S2. Fecal scoring system.

Stool Consistency	Scores
normal	1
soft to loose	2
loose to watery	3
watery, mucous, slightly bloody	4
watery, mucous, and bloody	5

Table S3. Primers for quantitative real time PCR

Target Gene[1]	Primer Sequences[2] （5'→3'）	Product Length
IFNG	F: TGATTCAAATTCCGGTGGAT<br>R: GCAGGCAGGAGAACCATTAC	166
IL17A	F: TTGGACCCTCCACCGCAA	308

	R: GGGTCTACTCCCCAGAAAGC	
IL17F	F: AGTTTCCTGCACAACGCAAG<br>R: GTCCCGGGTGATGTTGTAATC	285
IL6	F: CCAATCTGGGTTCAATCAGG<br>R: ACCCACTCGTTTGAGGACTG	241
IL1B	F: CATGTGTGCTGAAGGCTCTC<br>R: AGTGTCGGCGTATCACCTTT	173
Il-23R	F: GCAGCAGTGAGGAAGGAAGAC<br>R: GACCTGGTTCATGCCTTACG	102
TNFSF11	F: CAAGTAACAAGCCGGTAGCC<br>R: AGATGAGGTAAAGCCCGTCA	155
IL22	F: TCCAGGGAATCAATCAGGTGAC<br>R: AGCACATTCACGTTTCAGGGT	302
FABP1	F: AGATCAAGGCGGTGGTTCAG<br>R: CCCTTCGTCATGGTACTGGT	115
HMGCS2	F: AGGCAACACTGACATTGAAGGC<br>R: AGTGCGTAGCGACCATCCCA	117
RXRG	F: TGTTAGCCCAAGTTGAAGGGA<br>R: CGATGGACTCATGGACGTGG	191
CRABP1	F: ACTCATCCTGACGTTTGGCG<br>R: TTACTGGGGAGACATCGGGA	190
CRABP2	F: AGAGCTGATCCTGACCATGAC<br>R: GAACCCCCAGAAGTGACTGG	168
β-actin	F: TCTCTTCCAGCCTTCCTTCCT<br>R: GTGTTGGCGTAGAGGTCCTTG	111

Note: [1]*IFNG* = Interferon Gamma; *IL17A* = Interleukin 17A; *IL17F* = Interleukin 17F; *IL6* = Interleukin 6; *IL1B* = Interleukin 1 Beta; *IL-23R* = Interleukin 23 Receptor; *TNFSF11* = Tumor Necrosis Factor Superfamily Member 11; *IL22* = Interleukin 22; *FABP1* = Fatty Acid Binding Protein 1; *HMGCS2* = 3-Hydroxy-3-Methylglutaryl-CoA Synthase 2; *RXRG* = Retinoid X Receptor Gamma; *CRABP1* and *CRABP2* = Cellular Retinoic Acid-Binding Protein 1or 2. [2]F = forward; R = reverse.
